# In comparative perspective: The effects of incarceration abroad on penal subjectivity among prisoners in Lithuania

**DOI:** 10.1177/1477370817726716

**Published:** 2017-09-13

**Authors:** Gavin Slade, Rūta Vaičiūnienė

**Affiliations:** University of Glasgow, UK; Law Institute of Lithuania, Lithuania

**Keywords:** Eastern Europe, migration, prison, reform, subjectivity

## Abstract

This article looks at how global flows of people and policies affect penal subjectivity among prisoners in Lithuania. Those who had previously been incarcerated abroad perceive their punishment in Lithuania’s reforming penal system in comparative terms. We find that international prison experience may either diminish or increase the sense of the severity of the current punishment. Respondents often felt more comfortable in a familiar culture of punishment in Lithuania that emphasizes autonomy and communality. Moreover, internationalized prisoners perceive prison reform emulating West European models as a threat to this culture and are able to articulate comparative critiques of this reform and contest its effects.

## Introduction

The question that guides this article is as follows: how has heightened mobility of people and penal policies in Europe shaped not only the prison societies of migrant-receiving countries in the rich West and North but also the migrant-sending countries of the poorer East and South? Through a qualitative analysis of interviews with former migrants from Lithuania who have been incarcerated abroad and are now in the Lithuanian prison system, the article aims to discuss this question. The article shows that in Lithuania understandings of punishment are framed in comparative and international terms. This is for two reasons; firstly, the high prevalence of former migrants in prison who have been incarcerated abroad, and, secondly, the effects on prisoners of large-scale reform of the Lithuanian penitentiary system towards a ‘European’ model. To make sense of these factors, the article frames respondent narratives in the growing bodies of literature in criminology concerning crime and migration in the emerging criminology of mobility (Aas and Bosworth, 2013; Bosworth, 2012; Bowling, 2013; Kaufman, 2014; Stumpf, 2006; Weber and Pickering, 2006), as well as work on the global flow of criminological expertise and policy (Bowling and Sheptycki, 2012; Jones and Newburn, 2002; Melossi et al., 2011; Sparks and Newburn, 2002).

The article argues that international flows of both prisoners and penal policies has implications for the emerging work on penal consciousness or penal subjectivity, which addresses the variability in the ways in which punishment is experienced (Crewe, 2011, 2012; Sexton, 2012, 2015; Hayes, 2016). The key claim made in the article is that penal subjectivity – the meanings, orientations and understandings of punishment – is no longer shaped only by the penal power of the given state in which a particular prison is located. Instead, the perceptions of the severity and salience of punishment are becoming internationalized ‘in a world of global mobility’ (Bosworth, 2012: 126).

The article is structured as follows. Firstly, we provide a discussion of the literature and suggest certain gaps that this article aims to speak to. We then give an overview of the Lithuanian case, which establishes that Lithuania is a high emigration country that is undergoing major penal reform. The article then produces an analysis of prisoner narratives concerning their punishment in Lithuania and the comparative perspective these narratives offer. Finally, we discuss perceptions of Lithuania’s prison reform. We show that prisoners use a comparative lens to understand this; they feel that reforms conducted in the name of becoming ‘European’ often do not produce the same outcomes as in the Europe that the prisoners are all too familiar with. In conclusion, we suggest that the internationalization of penal subjectivity might be of interest to criminologists working in a range of subfields.

## Globalization and penal identities

A fast-growing literature within criminology concerns mobility, migration and criminal justice responses to these (Aas and Bosworth, 2013; Bosworth, 2011, 2012; Kaufman, 2014; Melossi, 2003; Stumpf, 2006; Weber and Pickering, 2006). This literature convincingly argues that punishment and criminal justice logics have become central to immigration control, in turn reshaping understandings of belonging and citizenship. As Kaufman (2014) writes, the basic premise of the criminology of mobility is that globalization has altered the relationship between punishment and identity. In an age of globalization, punishment exacerbates differences of race and ethnicity, reconfiguring gendered identities and their relationship with nationality and citizenship. The writing in this area focuses on the reproduction of global inequalities and the effects on identity of those ‘othered’ by exclusionary measures such as deportation regimes, immigration removal centres and the differential management of foreign nationals in prison. Related to this, some studies also attempt to explain the increasing proportion of foreign prisoners in the prison populations of Western Europe, linking this to political economic and institutional factors (De Giorgi, 2010; Lacey, 2008; Wacquant, 1999).

A further body of literature, somewhat separately, charts the effects of globalization on the circulation of criminological ideas, technologies and ‘expert systems’ (Giddens, 1990). Notwithstanding the embedded cultures of punishment that exist in national contexts (Garland, 1993; Nelken, 2011; Smith, 2008), diffusion and transfer across jurisdictions is commonplace whether in policing strategies, prison design or sentencing policies (Bowling and Sheptycki, 2012; Jones and Newburn, 2002; Melossi et al., 2011; Wacquant, 2009). Such diffusion is often accompanied by internationalized slogans such as ‘zero tolerance’, ‘no frills prisons’ or ‘broken windows’. Although many studies point to the international spread of US approaches to criminal justice (Downes, 2001; Jones and Newburn, 2002; Karstedt, 2002), the European Union (EU) has also endeavoured to create a unified ‘area of justice’ that supposes a degree of convergence and uniformity in crime control and punishment (Baker, 2013; Snacken and Van Zyl Smit, 2009).

Despite these growing bodies of literature, Bosworth (2012: 125) writes that ‘many researchers in [the punishment and society] field have … been slow to recognize the impact of globalization on structures, practices and *experiences* of punishment’ [our italics]. The gap in our knowledge concerning shifting penal experiences in conditions of global flows of people and ideas is one of the key objects of study for the criminology of mobility. This article offers a case study from a non-Western jurisdiction. The case of Lithuania shows that subjective experiences of punishment in predominantly migrant-sending countries are also changing significantly owing to global mobility. Moreover, migrant-sending countries are most likely to be poorer and positioned on the receiving end of flows of global expertise, including reform pressure from the EU and policy promotion from political and civil entrepreneurs.

The Lithuanian case demonstrates how flows of people and ideas impact penal subjectivity or penal consciousness – the experience of something as punishment and the meaning ascribed to that punishment (Sexton, 2015). For socio-legal and jurisprudential theorists, penal subjectivity concerns measuring the variable degree of individual suffering at the hands of the state so as to establish the justness of a particular sanction (Grabosky, 1978; Hayes, 2016; Kolber, 2009). The pains of imprisonment are not the same for everyone and are experienced subjectively across a range of dimensions (Crewe, 2011, 2012). In this vein, Lori Sexton, following prior distinctions concerning the law as it is in action compared with on paper, has tried to understand the lived realities of ‘punishment in action’ as against ‘punishment on the books’ (Sexton, 2015: 117). She argues that the severity, or the intensity, of punishment and the salience, or the prominence, of the punishment in everyday life differ from prisoner to prisoner. The degree to which a prisoner feels the intensity and salience of punishment depends on ‘the punishment gap’. This refers to the degree to which each individual’s expectation of punishment and the actual experience of punishment diverge. Expectations, for Sexton, are determined by vicarious knowledge and comparison with other prisoners’ punishment as well as prior experience.

The article aims to build on Sexton’s theory concerning the variability of penal subjectivity and the role of comparison in penal suffering. We develop the theory by arguing that such comparison can have an international dimension under conditions of globalization. Our two central propositions are, firstly, that international experience of prison enables comparison of punishment across jurisdictions, differences in these experiences will affect expectations and hence the punishment gap; and, secondly, that international experience of prison provides the basis to critique and resist penal policy innovation as well as to adjust expectations of these innovations’ effects on experiences of punishment. Lithuania provides a critical case study to explore these propositions. Below we introduce the case study and show why Lithuania is a particularly appropriate case for understanding the penal impact of global mobility in a non-Western European context.

## Lithuania: International emigration and domestic prison reform

Lithuania is a country of 3 million people that, apart from Latvia and Estonia, is the only EU member state to have been a Soviet republic. Like many former communist states, Lithuania has been a net emigration country since 1990. It is estimated that 825,000 people have left the country since that time (Europas migracijos tinklas, 2013). However, emigration is often not permanent. Immigration into Lithuania mostly comprises returning migrants and their number steadily increased through the 2000s; it has seen a significant upward trend since 2010 and reached its highest point in 2014 when 24,294 people returned, up from 5553 in 2004.

Lithuanians have migrated to many countries. One of the main destinations has been the UK. Rasinger’s (2010) study of UK press coverage has shown that Lithuanians have been portrayed as quintessential criminal migrants. Lithuanians are over-represented in the UK prison system: they comprise 2.8 percent of the UK’s foreign-citizen population yet make up 4.9 percent of the UK’s foreign prisoner population (Giannangeli, 2013). Siegel’s study of itinerant criminals in the Netherlands showed that Lithuanians featured the most prominently in police statistics for migrant crime, followed by Poland, Bulgaria and Romania (Siegel, 2014, 2015). This article is not concerned with trying to understand why some Lithuanians turn to criminality once abroad. These statistics from the Netherlands and the UK merely establish that Lithuania can fairly be portrayed as an EU member state that has a highly mobile population and that some Lithuanians indeed fall foul of the law in some receiving countries, experiencing life in prison in those countries.

In Lithuania itself, the penitentiary system is in the throes of reform driven by international expertise. At 315 prisoners per 100,000 of the population, the Lithuanian penitentiary system proportionately holds the most prisoners in the EU (International Centre for Prison Studies, 2014). As a comparison, Germany, one of the main destination countries for Lithuanians, holds 76 prisoners per 100,000 of the population. The majority of the 9700 prisoners in Lithuania are held in houses of correction; these were known in Soviet times as correctional colonies (Sakalauskas, 2014).^1^ Correctional houses occupy large areas that contain both industrial and living ‘zones’. Convicted prisoners are held in dormitories, rather like barracks, housing between 10 and 20 people and sometimes more. Two or three dormitories make up a prisoner detachment, which in Soviet times would have worked shifts together. Prisoners are able to leave their dorms for almost the whole day, walking in their ‘local sector’, a separated area containing a set number of dormitories and communal areas. At night prisoners are able to go out into the corridor because bathrooms are outside the dorms. The majority of prisoners do not wear uniforms, they have access to a shop with no limit on their daily spend, and they can have personal effects such as games consoles. They are allowed to use computers for up to three hours a day, though with no Internet access.

Staff supervision is relatively minimal, with ‘controllers’ or guards doing the rounds through the local sectors at various times in the day. The large grounds and open space of the zone, combined with the relatively self-regulated prison life, mean that illicit mobile phones and drugs come over the wall and are rife in Lithuanian prisons. Some informal legacies of the Soviet period are also still present; there is a relatively rigid caste system in place. Subcultural codes frame social practices and interactions. Individual prison administrations still employ officers as ‘operatives’ who recruit prisoners to become informers, a very Soviet version of dynamic security.

Lithuania has taken a soft and slow approach to reforming the collectivist aspects of the Soviet-style colonies. The country signed up to a number of international obligations as part of its integration into the EU, NATO and the Council of Europe. The carrot of EU membership, which came about in 2004, stimulated further reforms. A new Lithuanian Code of the Execution of Penalties was introduced in 2003, as was a new Criminal Code (Sakalauskas, 2014). Expert commentators believe that since that time reform processes have stalled despite demands from EU institutions to reform the Soviet remnants in the system.^2^ The condition of Lithuanian correctional colonies has been used as grounds to appeal extradition. Violence in the system, owing to the open nature of the colonies and the system of castes that still exists, has brought successful litigation against the government by prisoners in the European Court of Human Rights (see, for example, *Tautkus vs. Lithuania*, 2009).

Yet, there are still intentions to reform the system. Much of this comes with international expertise. Sweden and Canada have been instrumental in assisting with alternatives to prison, improving juvenile justice and creating a probation service. With the help of Norwegian financing, for example, halfway house open colonies are being implemented. However, the real essence of these reform plans is a move to a ‘cell system’. Currently this is planned for completion in 2022, though much scepticism remains. The plan involves a major period of new building and renovation of old buildings. The aim is to move prisoners out of barracks and provide closed living and social spaces along West European lines. Principles and practices of ‘dynamic security’, with the assistance of Norwegian funding, will replace the guards on prison fences (Prison Department of Lithuania, 2015). The process of closing off the wide spaces of the colony has been in progress since Lithuanian independence. Prison administrations have continually erected physical barriers to produce higher numbers of so-called ‘local sectors’ with fewer prisoners in them in order to undermine the caste system. Some sectors are designated to hold people in the higher caste, who live by Soviet criminal traditions, in an attempt to isolate and control the influence of this form of extra-legal prison governance.

Lithuania then represents a country affected intimately by the globalized mobility of people and policies. The article now seeks to understand how these processes affect Lithuanian prisoners’ penal subjectivity. We will look at the internationalization of the penal experience of prisoners in Lithuania and how this affects the gap between expectations and realities of punishment. We then turn to how mobility produces competing assessments among prisoners of internationally informed penal reform efforts and understandings of what a ‘better’ system should look like. Before we turn to examine these issues, we briefly discuss the data that the subsequent analysis is based upon.

## Data and methods

The data for the following sections come from a qualitative study of prison social life during reform in selected post-Soviet countries. Lithuania was one of the case studies in this project. In total, 56 in-depth interviews were carried out by the authors in Lithuania: 5 of these were with experts (current and former policy makers and practitioners), 13 were with staff, including prison guards, social workers and a prison director, and 38 were conducted with prisoners across three penal institutions (all correctional houses) during a one-month period. The three institutions were Alytus and Marijampolė, correctional houses mainly for recidivists, high-risk offenders and drug addicts, and Pravieniškės, mainly holding first-time offenders. Interviews lasted 1 hour in most cases and were conducted in Lithuanian or Russian.

Questions focused on social relationships in the context of reforming Soviet-style barracks and ‘local sectors’. Questions were also asked about knowledge of government plans to construct a cell system and prisoner views about this. Upon analysis of the interviews we found that 39 percent (*n* = 15) of prisoner respondents had served a sentence abroad in a total of 11 different countries; 4 had experience of prison in multiple countries other than Lithuania. Every one of the 15 had spent time in a prison in another EU country. Three also had experience of other post-Soviet countries’ prison systems, and two had been in prison during Soviet times. This variation in experience is shown in Table 1.

**Table 1.** The various countries in which respondents had had prison experience.

Prison experience in …	Number of respondents
Germany	4
UK	3
Spain	3
Estonia	3
Russia	2
Italy	1
Belarus	1
Latvia	1
Sweden	1
Belgium	1
France	1

This sample of prisoners with international experience was random; we did not select prisoners with such experience intentionally. However, we do not believe that the percentage of those with prison experience abroad in our sample is somehow representative of the percentage of such prisoners in the broader Lithuanian prison population. There are a number of selection biases. Firstly, prisoners were selected with the help of social workers, and the more assertive and talkative prisoners often self-selected to be interviewed. These types of qualities might also be important for a decision to emigrate. Secondly, the average age of our sample (33 years) was slightly older than the total prison population average (around 29 in 2012), and this may also have skewed the number of respondents we had with foreign prison experience. We do not include respondents with no foreign prison experience in our analysis. However, these respondents also had vicarious knowledge of foreign prisons from others.

Lithuania is a very particular case that might not be straightforwardly generalizable. It is a post-Soviet republic and therefore has a more distinct culture of punishment than other European jurisdictions. It has a particularly high level of out-migration (Europas migracijos tinklas, 2013). Yet, in terms of migration Lithuania is far from unique, particularly in comparison to the countries of Southern and Eastern Europe. Thus, although the following analysis is of a single case, the insights might be applicable to other places outside Lithuania.

In what follows we focus mainly on prisoners’ comparisons between Lithuanian and West European prisons; we include Estonia in this latter category because its prison system has been reformed to a cell type and no longer maintains Soviet-era colonies. On these grounds, Latvia, Belarus and Russia do not qualify as ‘European’ but these cases are still brought into the analysis where relevant and for comparative purposes. When providing excerpts from interviews, we give respondents a changed name, state the prison where the interview took place and the foreign prison systems of which the respondent had experience.

## Comparative perspectives and penal subjectivity

Respondent accounts demonstrated that the gap between expectations and the reality of punishment was indeed mediated by the comparative experience of prison abroad. However, the gap could be widened or shortened by the experience and this varied according to the individual or the particular punitive referent – the given object that elicited a sense of being punished (Sexton, 2015) – in question. For some, the prisons of Western Europe were seen as ‘luxury’ in terms of material conditions, privacy and security. For others, prison in Lithuania fitted with cultural expectations of punishment, easing the experience compared with what they had experienced abroad. Moreover, the architectural style of the Soviet colony provided greater communality and freedom. This section will highlight both types of perception, drawing on the narratives of the prisoners themselves. The comparative narratives that the prisoners told revolved around certain central concepts that frame the discussion below. These are: dignity, autonomy, communality, order, privacy and identity.

Respondents compared their treatment by staff and expressed a sense of greater inhumanity and indignity at their treatment in Lithuania when compared with abroad. In other countries, respondents reported that staff had behaved in a more ‘cultured’ manner. In Lithuania, some respondents mentioned being viewed and addressed as non-human. As one respondent, who had been transferred from Estonia to Lithuania on his own request, explained:

When I got here [Lithuania] I almost went crazy, I thought I’d landed back in the Soviet Union! The beds are dirty. And the guards were always shouting. The whole night I was sitting there thinking, we had it was all so clean and well appointed [in Estonia]. And I thought, fuck me, why did I come here?!

(Petras, Pravieniškės – Estonia)^3^

Expectations about how to be treated, based on experiences abroad, were wide of the reality and hence exacerbated the punishment gap once back in Lithuania. Respondents also drew comparisons between management techniques and practices of formal governance of the prison population and how these affected their sense of being punished. One respondent who had been in prison in Belarus and Sweden found Belarus to be more comparable to Lithuania precisely in the methods of managing the prisoners and everyday routine:

I understand it now. The type of prison you have, the type of state it is … Sweden, well, of course, it’s heaven you can say. It’s so calm! Whereas here, [t]here are some things from Soviet times, like the roll call. Why force people out for a roll call when you have every opportunity to count them normally like they do in Europe?

(Valery, Alytus – Belarus, Sweden)

After the conditions of prisons in Western Europe, perhaps the main element of disillusionment was the material conditions in the often decrepit and old colonies of Lithuania. The food was a point of contention, as was cleanliness. Owing to the poor food, conditions in the dormitories and distant and hostile relations with staff, prisoners in Lithuania, as in Soviet times, maintain a sense of self-reliance. Yet, while this produced relative deprivation in relation to material conditions and well-being, self-help and autonomy could also be important sources of dignity that some respondents felt had been denied to them when abroad. Thus, some suggested they had lost a sense of independence while abroad that could be rediscovered in the colony in Lithuania.

In Germany … nobody makes decisions themselves, they are not taught to do this … if someone has an issue they shout to the guard… I mean, they are not capable of resolving their own issues. And when it’s like that, when people tell you exactly how to live then when you get out it’s going to be even harder to live isn’t it? Because you’ll be used to having people doing things for you… But [in Lithuania] you can go outside get a book, watch TV, just sit and chat.

(Jonas, Alytus – Germany)

As this respondent suggests, the cell systems of prisons in Western Europe did not necessarily fit with what Lithuanians expected from punishment. A cell system for Lithuanians, as for other post-Soviet nationalities, is associated with Soviet use of cells. The Soviets used cells exclusively for police investigation units, remand or the segregation unit in colonies. Thus, cells represent extreme dependence, vulnerability, extra punishment, and a sense of loss of dignity and autonomy. Transfer to a colony represented a move to a more open space, dormitory living, heightened interaction and greater autonomy:

You can walk around in this big sector, you can go down to the yard … somebody is watching a movie or playing dominos in the sector … You take some fresh air or you go to the gym or visit somebody for a cup of tea. It is a different situation when you are in a closed cell.

(Vytautas, Alytus – England)

Some respondents, arriving in Lithuanian colonies from cell systems, describe a sense of relief and the colony as ‘small freedom’. The desire for the perceived freedom and autonomy in a colony moved two respondents to request transfers back to Lithuania. One respondent decided he wanted a move back to Lithuania from Estonia after discussing it with other Lithuanian prisoners there because, as he put it, ‘[Lithuania] still has the camp system, it’s open, you can go out in the yard, use telephones, everything is possible’ (Petras, Pravieniškės – Estonia). A separate respondent did not like the reformed cell system of Estonia because of the restrictions on activity and movement. He was transferred back to Pravieniškės, a large colony (1400 prisoners) in central Lithuania, even though family members remained behind in Estonia:

In Estonia I was inside for two and a half [years] – in a cell … it’s enough! I asked to be moved here because this is a colony, I live here like I’m at home … Now I want to walk and walk and walk. It’s freedom here. It is. I feel freedom.

(Gintaras, Pravieniškės – Estonia)

For both of these respondents, the punishment gap was narrowed significantly by the familiarity of Lithuanian punitive practices compared with those elsewhere. These two respondents had similar histories of transfer from the same place, Estonia, but very different reasons for wanting the transfer. Gintaras was particularly athletic and simply felt that the colony suited him better to be active. He reported that he was something of a loner and not interested in involving himself in the subcultural caste system or gangs that are prevalent in Lithuanian colonies. Although he wanted the freedom of movement of the colony, he did not want the interaction. The other respondent, Petras, lived by the subcultural ‘understandings’ of Soviet times, supported the caste system, but felt he was better able to express this in the interactive everyday life of the Lithuanian colony rather than in Estonian cells where the subculture had weakened. Thus, the sense of closing the punishment gap for one was about the freedom to be active, for the other it was about the ease of achieving social respect and standing in a familiar context. Both of these respondents worked with an implicit comparative understanding of what punishment was to them as Lithuanians while in prison abroad. This understanding grounded their justifications for requesting transfers to Lithuania.

Although freedom and autonomy may be one advantage of the Lithuanian colony compared with international experiences, order and privacy are not so well guaranteed and appear to exacerbate the punishment gap after prison abroad. In the communal conditions of the colony, order depends on strong subcultural understandings and informal institutions. Yet, deliberate problematizing and targeting by prison administrations has significantly weakened these structures. In Lithuania, a decline in the generalized Soviet inmate code has led to an increase in the presence of city- or region-based gangs who exist in wary equilibrium of each other. Moreover, drugs are a big problem for the maintenance of order but appear to be highly prevalent and largely un-policed in the autonomy of the dormitory and local sector. Thus, many respondents found, relative to their experiences abroad, that order was not guaranteed in such a system:

Sometimes [other prisoners] will just take things off you, your clothes … There are people in here who have been sitting two or three years without meeting anyone, they never come out, they close themselves off completely from this system. There are so many like that. Out of fear.

(Daumantas, Alytus – Russia, Germany, Italy, Estonia)

Staff respondents corroborated this claim. They reported that some prisoners deliberately broke rules to engineer a move to the segregation unit in order to be removed from their dormitories and placed in a cell. Milhaud and Moran (2013) report similar findings in Russian prisons operating colonies similar to those in Lithuania. Violence is difficult to measure but it is certainly a problem that has been highlighted in a number of cases that relate directly to the control that the prisoner caste system still exerts. Some cases have gone to the European Court of Human Rights (ECHR), where the Lithuanian state has been found culpable for failing to provide protection from gangs or caste-based victimization (for example, *Tautkus vs. Lithuania 2009*; Human Rights Europe, 2015).

Conflict can stem from a lack of privacy. This is clear to any visitor to a colony dormitory, where prisoners clearly try to create their own space with bed sheets and towels only to have these taken down when the controllers come around for a check-up. Lack of privacy and overcrowding have been other substantial grounds for cases that have gone to the ECHR (see *Mironovas and others vs. Lithuania 2015*). Although the ECHR in some of these cases notes the poor conditions within the dormitories, it also recognizes that the free movement and space provided by the colony during the day can offset the ill effects of the poor indoor environment. Nevertheless, in such a situation, with a deteriorating subculture and a weakening of the attendant normative strictures on the use of violence, frustrations with co-inhabitants can boil over. In cases of conflict, prisoners may simply move of their own accord during the day to spend time in other dorms, coming back to their ‘detachment’ only to sleep. In some respects, this also demonstrates a level of autonomy and freedom of association. However, other prisoners or gangs may also force such movement. For prisoners with international experience this compared badly with their experience abroad, where they were moved in the event of conflict and generally had less interaction.

In Germany, for example, you can say, I don’t like you and don’t want to see you and they will move you. It’s written in the law there that you can choose … but here every day you see that same person … and you feel that tension every day and you can’t get out of it. [The staff] will tell you, you have to stay here, they can do it on purpose even, as an extra punishment.

(Daumantas, Alytus – Russia, Germany, Italy, Estonia)

Many respondents made this claim of extraordinary and informal punishment through forcing bad company on troublesome individuals. Thus, those who, while abroad, had felt relatively empowered in choosing whom they shared physical space with keenly felt a lack of privacy back in Lithuania. Yet, for some, considerations of privacy versus autonomy were not as important as a general sense of familiar personal identity when back in Lithuania. In line with findings from studies of foreign nationals in UK prisons and detention centres (Bosworth, 2011; Bosworth and Slade, 2014; Kaufman, 2014), Lithuanians in prison abroad often felt that their status as non-citizens stigmatized them and created insecurities they simply did not feel back in Lithuania. Furthermore, for many the diversity of prisoners in Western prisons, highlighted by scholars of the criminology of mobility, created problems of integration, belonging and mutual recognition and respect. This was especially true in comparison with the simpler caste system and ethnic homogeneity of Lithuanian prisons.

Thus, one respondent had wanted to be removed from UK prison as quickly as he could, despite his wife living in the country. The same forces of globalization and mobility that had brought him to the UK had created a diversity and cultural mix that he was uncomfortable with. He preferred the relatively straightforward ethnic relations of Lithuanian colonies comprising mainly Lithuanian prisoners alongside minority groups of Poles and Russians.

In England the judge … said that there is a lot of talk about the conditions [in Lithuanian prisons] and so maybe they will not need to deport me to Lithuania. But I wanted to come back to Lithuania as soon as possible … because [in the UK] there are, you know, Muslims and it is not acceptable for me to sit with those kinds of people … I do not want to be inside with black people or Muslims.

(Vytautas, Alytus – England)

Compared with Lithuania, racial and ethnic mixing was experienced as part of the punishment for this respondent, threatening and diluting any sense of identity other than a foreign national prisoner. Social relations inside the Lithuanian colony were easier to negotiate and on the respondent’s return home the punishment gap, on this measure, was shortened. The issue of identity then, along with the more familiar philosophy and practices of punishment, was an important factor in mediating the subjective experience of prison upon return home.

There are then ambiguities in the subjective experience of punishment that incarcerated returned migrants feel. These ambiguities stem from the fundamental differences in the cultures of punishment in Western Europe compared with the post-Soviet region, born out of two different visions of penal modernization in capitalist and communist societies. These differences relate to dignity, autonomy and communality, as well as to privacy and order. Moreover, familiarity and national identity also shaped subjective experiences of the current confinement. Respondents discussed their imprisonment in Lithuania in all of these terms yet comparison was a key frame of reference for understanding their punishment’s severity and salience and their ability to find dignity and respect now back in their country of origin. Thus, penal mobility can have variable effects on the gap between expectations and actual experiences of punishment.

We now turn in greater detail to this issue of legitimation and contestation in the Lithuanian penitentiary system with particular reference to the issue of prison reform. As discussed previously, the reform to a cell system away from the colony form of punishment is ongoing in Lithuania and there has been a great deal of international influence on reform processes in the criminal justice sector from the EU, Scandinavian countries and Canada. Thus, in Lithuania internationalized prisoners meet internationally driven penal reform projects in their everyday environment. The next section looks at how they make sense of such projects comparatively.

## Comparing and contesting reform

All respondents were aware of the planned reform project to change to a cell system by 2022 and gave opinions on it. Most were sceptical about the possibility that it would ever be fully implemented. The same concepts of dignity, autonomy, communality, order, privacy and identity frame discussions about the type of prison that Lithuanian prisoners expect and the type of reform they find acceptable. Many prisoners who possess international experience are ambivalent about the reforms, preferring elements of the familiar Soviet-style culture of punishment, finding some European reforms inappropriate for the Lithuanian reality, at the same time as recognizing the potentials benefits of Europeanization.

Respondents reported that the aims of the reforms did not affect their sense of punishment as much as the manner in which reforms were carried out. In particular, piecemeal attempts to move towards European norms were felt to increase penal impact and suffering. This related in particular to reforms that reduced autonomy and independence such as reductions in allowances for personal belongings, spending restrictions in the prison shop, or the reduction in size of local sectors to reduce the prominence of informal governance through the caste system. Once again comparing with conditions in Western Europe, respondents reported that such reforms were not carried out with the necessary and corresponding changes to compensate them. Attempts to limit what could be bought at the prison shop produced a countrywide hunger strike in 2012 involving 80 percent of the prison population.^4^ The shop limit was lifted, but there is a constant readiness to fight other, similar, reforms:

Now they are saying they can … limit what we can bring into the zona [colony] ourselves. I can bring 30kg in and they say – ‘what do you need it for?’ I’m saying I need it; I need a coat against the cold because they give you nothing here. And they say they are doing it like in Europe, but in Europe they give you everything! Blankets and underwear, socks, you don’t need anything there, that’s why they can do it … Then they say: ‘You buy so much from the shop.’ And they say ‘in Europe you can only buy sweets and biscuits at the shop’; well of course because they provide decent food there.

(Daumantas, Alytus – Russia, Germany, Italy, Estonia)

The main theme in the narratives concerning these reforms was concern over a shift to a dependent position and reliance on prison regimes after the relative autonomy of the colony system. As the prison administration closed down avenues for self-reliance, prisoners would be slowly closed in and forced into dependency on the regime. The pains of imprisonment were being reconfigured by the reform. A clear example concerned visiting rights. Visits in Lithuania are permissive compared with those in other jurisdictions. Conjugal visits are possible, visiting times are longer and greater freedom of movement is permitted during visits. One respondent, Antanas (Pravieniškės – England), compared this situation favourably with the UK, where conjugal visits are not permitted. Respondents were scathing about any changes to the rights to visits in the name of European reform. Yet, the reform was already ongoing and there was a sense of foreboding in many narratives; the reform would only cause greater suffering, conflict and resistance. Moreover, as one respondent mentioned, such suffering could be justified by referring to how things are done in Europe:

They don’t want to manage us, no – they want to slowly put us in cells so they can control each person, close them in. They want to take away our visits, because they can say that visits don’t happen in Europe. To take it away though, we will riot, hunger strike and so on, in every camp and prison in Lithuania.

(Jonas, Alytus – Germany)

Increased violence and disturbances during prison reform have been recorded in other post-Soviet cases (Piacentini and Slade, 2015; Slade, 2015). The ability to organize resistance in such contexts is grounded in the complex forms of informal group governance that have developed in the open spaces of prison colonies such as those in Lithuania. Prisoners subordinate themselves to the informal norms and institutions created within these spaces more readily than they do to the administrative prison regime. As we have seen, such self-organization can be perceived as either increasing or decreasing the severity of punishment depending on the individual, creating order for some and insecurity for others. Staff respondents were candid that the cell-system reform in Lithuania, in part incentivized by high-profile cases of violence censured by the ECHR, was aimed at reducing informal governance forms by the caste system and gangs. Reforms have already shrunk the size of local sectors to make them more manageable. Those who profess strong affiliation with the criminal subculture – often referred to as ‘mafiosi’ by respondents – were separated and confined to their own local sector with high walls and situated far from the other inmates.

These reforms also produced conflicting narratives about respondents’ feelings of being punished. In a context where the penal system provides few opportunities to improve skills to aid reintegration in society upon release and provisions for resettlement are poor, the social life of the colony is seen by some respondents as a compensatory mechanism in which prisoners may nurture social skills that are useful upon release. Again, respondents with international experience expressed frustration that the provisions given to prisoners in other parts of Europe were not available to them at a time when reform would leave them ever more deprived. Space was a critical matter in this. The sense of space closing in, local sectors getting smaller and social circles narrowing also produced feelings that suggested reform was in fact widening the punishment gap. Some respondents reported that they tried to arrange a move into the most spacious sectors to maintain the communality that they felt was characteristic of the penal colony:

Other local sectors are smaller. We have greenness, grass and it gives you a lot, that. [In other] sectors … you go out and all that you can see is the sky and concrete. [We] can get a tan, lie down on the grass! It makes a difference.

(Vladimir, Alytus – Spain)

This was not true for everyone of course, and some who had been in prison abroad had got used to the privacy and welcomed the move to the cell system on these terms.

I liked being in a one-man cell [in Sweden]; you can be yourself, maybe some people wouldn’t like it, there’s less communication. But for me if they open the doors in the morning and close you in in the evening then in the evening you are dedicated only to yourself.

(Valery, Alytus – Belarus and Sweden)

Reform in Lithuania is a contested notion by those in the prison population who have spent time in prison abroad. Certainly, some prisoners rejected the cell-system reform completely based on their experience in other countries in Europe. However, most narratives were more complex: internationalized Lithuanian prisoners do not reject the move to a cell system absolutely, but feel that such reform is skewed towards changes that increase the pains of imprisonment without any of the ameliorating conditions of Western European prisons that would make such changes tolerable. Respondents often believed that notions of ‘Europeanness’ were employed by reformers to justify particular undesirable reforms, such as limits on spending in the shop or the allowances on personal belongings. Referencing a reform as ‘European’, ‘in line with EU norms’ or ‘to European standards’ uncritically implied that this was shorthand for quality or humaneness.

## Conclusion

This article suggests new avenues of enquiry concerning the relationship between globalization and punishment. Firstly, the criminology of mobility assumes that globalization is radically altering the interplay of punishment and identity. This article suggests that this proposition should be examined in cases outside of Western Europe. Our case study shows that many Lithuanian prisoners have experienced prison abroad and that this affects individual perceptions of punishment not only in terms of identity. Secondly, there is a growing literature on the global flows of criminological expertise, policy and reform packages but few studies directly link these to their effects on individual subjects. This article demonstrates that while Lithuanian politicians seek international expertise and foreign models to reconstruct the physical basis of the Lithuanian penitentiary system, internationalized prisoners are able to articulate comparative analyses concerning what they seek from these reforms in terms of identity, order, privacy, dignity, communality and autonomy. Thus, there is much more that could be said about the links between the international dimensions of penal reform and prisoners’ internationalized understandings of punishment.

As regards such understandings, we have utilized the concept of penal subjectivity (Sexton, 2012, 2015) for interpreting the narratives of prisoners who have been incarcerated in numerous countries. Sexton’s framework emphasizes the impact of comparison, vicariousness and intersubjective assessments of punishment on penal suffering. Building on this insight, this article suggests that international experience facilitates comparison, which in turn informs penal subjectivity and affects the punishment gap by adding an extra dimension to penal suffering.

Thus, this article makes the case that research that aims to reconceptualize the pains of imprisonment (Crewe, 2011), measure penal impact (Hayes, 2016) or map penal consciousness (Sexton, 2015) may find internationalization a confounding factor that requires scholarly attention owing to the increasing numbers of people with experiences of more than one culture of punishment. Moreover, Pallot et al. (2012) argued that, in the case of Russia, forcible movement while incarcerated was itself an additional pain of imprisonment. Prisoners in Europe will become increasingly mobile as intergovernmental agreements on transfer become active and prisoner consent will not be required. The effects of this on subjective experiences of punishment should be of interest to criminologists.

Finally, in Lithuania, both policy makers and prisoners perceive the evolution of the penal system in terms of becoming ‘European’. The internationalized prisoner body, however, does not accept this process uncritically. Unfortunately, prisoner voices are likely to remain relatively unheard. As has been argued elsewhere (Piacentini and Slade, 2015), many penal reform projects in the post-Soviet region have been carried out without any thought to a broader discussion of what prisoners expect from punishment culturally and how certain forms of collectivism might be desirable. Meanwhile, prison reform advocates in Western European countries argue for less individualizing, more open and communal prison designs (Hancock and Jewkes, 2011). Thus, while reform develops along opposite trajectories in different parts of Europe and the notion of ‘European penology’ becomes disputed, we suggest that, owing to mobility, prisoners themselves have become better able to assess the merits of these different philosophies and approaches. The incredible mobility that characterizes modern Europe may well be shifting understandings of penality from the inside, transforming local cultures of punishment, providing new bases of contestation – especially over reform – and filling prisons with comparative criminologists.

## Notes

1. Formally penitentiary institutions in Lithuania are called ‘correctional houses’ and contain almost all of the male prisoner population. There is only one actual jail in Lithuania with a cell system for remand prisoners and those on life sentences. When we use the term ‘prison’ in this article we are referring to Lithuanian correctional houses.

2. Interview with former deputy Minister of Justice of Lithuania 2003–6 and law professor, 15 July 2014, Vilnius.

3. All names have been changed in line with guarantees of anonymity.

4. Interview with former deputy Minister of Justice of Lithuania 2003–6 and law professor, 15 July 2014, Vilnius.
